# Attitude and awareness of medical and dental students towards collaboration between medical and dental practice in Hong Kong

**DOI:** 10.1186/s12903-015-0038-2

**Published:** 2015-05-02

**Authors:** Shinan Zhang, Edward CM Lo, Chun-Hung Chu

**Affiliations:** Faculty of Dentistry, The University of Hong Kong, Hong Kong, China

**Keywords:** Medical-dental collaboration, Attitude, Awareness

## Abstract

**Background:**

Medical-dental collaboration is essential for improving resource efficiency and standards of care. However, few studies have been conducted on it. This study aimed to investigate the attitude and awareness of medical and dental students about collaboration between medical and dental practices in Hong Kong.

**Methods:**

All medical and dental students in Hong Kong were invited to complete a questionnaire survey at their universities, hospitals and residential halls. It contained 8 questions designed to elicit their attitudes about the collaboration between medical and dental practice. Students were also asked about their awareness of the collaboration between dentistry and medicine. The questionnaires were directly distributed to medical and dental students. The finished questionnaires were immediately collected by research assistants on site.

**Results:**

A total of 1,857 questionnaires were distributed and 809 (44%) were returned. Their mean attitude score (SD) towards medical-dental collaboration was 6.37 (1.44). Most students (77%) were aware of the collaboration between medical and dental practice in Hong Kong. They considered that Ear, Nose & Throat, General Surgery and Family Medicine were the 3 most common medical disciplines which entailed collaboration between medical and dental practice.

**Conclusion:**

In this study, the medical and dental students in general demonstrated a good attitude and awareness of the collaboration between medical and dental practice in Hong Kong. This established an essential foundation for fostering medical-dental collaboration, which is vital to improving resource efficiency and standards of care.

**Electronic supplementary material:**

The online version of this article (doi:10.1186/s12903-015-0038-2) contains supplementary material, which is available to authorized users.

## Background

Oral Health is an integral part of general health [1]. It is vital to overall health and often associated with other systemic conditions [2]; thus, medical-dental collaboration is essential for people’s well-being. Interprofessional collaboration is a ‘partnership between a team of health providers and a client in a participatory collaborative and coordinated approach to shared decision making around health and social issues [3]. It is a process in which different professional groups work together to improve health care. Interprofessional collaboration achieves greater resource efficiency and improves the standards, comprehensiveness, and continuity of care by reducing duplication and gaps in services [4]. This improved professional cooperation between medical and dental practitioners may benefit all parties and better educate the public.

Most medical doctors and dentists are trained locally in Hong Kong [5,6]. The University of Hong Kong and The Chinese University of Hong Kong each produced about 150 medical doctors per year through a 5-year undergraduate program [7]. There is one dental school in Hong Kong training about 55 dentists per year. The medical doctors and dentists are trained as distinct professionals and have separate responsibilities. Dentists often focus on the diagnosis and treatment of oral diseases and may overlook other health problems [8]. Likewise, doctors may fail to notice their patients’ oral health problems. Enhancing health care services through interprofessional collaboration between medical and dental practitioners is therefore important, even essential [8].

Yet limited literature has been published on it. An article was published after the First Systemic Health Round Table Discussion to advocate for better medical-dental collaborative practice [9]. The article pointed out a long-standing segregation between medical and dental professionals that hindered multidisciplinary care in certain systemic conditions. Interprofessional collaboration includes communication and decision-making, enabling a synergistic influence of grouped knowledge and skills [10]. A proficient collaboration between medical and dental practice not only fosters people’s health but provides cost-effective, multidisciplinary care for patients. The purpose of this study is to investigate the attitude and awareness of medical and dental students towards collaboration between medical and dental practices in Hong Kong.

## Methods

### Recruitment of medical and dental students

All medical students in the University of Hong Kong and the Chinese University of Hong Kong and all dental students in the University of Hong Kong were invited to participate in a questionnaire survey. The sample size was 1,857. Written informed consent for participation in the study was obtained from participants. With the permission of the university professors, research assistants distributed questionnaires to the students after lessons in lecture theatres. Since senior medical students in years 4 and 5 receive clinical training in small groups in hospitals and stay at residence halls, the assistants at their halls invited them to join this study with the permission of the hall office supervisors. The students completed the questionnaire under the supervision of research assistants, who collected the completed questionnaires immediately.

### Questionnaire design

The questionnaire was designed to collect medical and dental students’ demographic information and assess their attitude towards the research topic. The demographic information included curriculum (medical or dental), year of study (junior year 1–3 or senior year 4–5), age (below 21 or 21 and above), gender (male or female), relationship with a regular family physician (yes or no) and relationship with a regular dentist (yes or no). The last variable referred to those students whose last dental check-up was within the last 12 months. Even if students had attended a dental clinic for treatment, those whose last dental check-up was more than a year ago were considered non-regular dental attenders. Eight questions (yes or no) were asked to assess attitudes about the collaboration between medical and dental practice. One question (yes or no) was asked about awareness of the collaboration between medical and dental practice. These nine questions were adapted from Hendricson and Cohen [11] and Migliorati and Madrid [12]. A pilot study was carried out with a group of para-medical undergraduate students who were asked to comment on the questions. The wording of the questions and the answer options were then modified to improve their clarity, avoid ambiguity and enhance comprehensiveness. The final questionnaire consisted of 16 questions (attached as Additional file 1). It included the general profile of the participants (Question 1 to 6), attitude towards the collaboration between medical and dental practice (Question 7 to 14) and awareness of the collaboration between medical and dental practice (Question 15). For those students aware of the collaboration between medical and dental practice, a follow-up question was asked to indicate their perceived linked of dentistry and the medical disciplines recognized in Hong Kong. The disciplines were i) accident & emergency services; ii) family medicine; iii) psychiatry; iv) cardiothoracic surgery; v) obstetrics & gynaecology; vi) radiology; vii) clinical oncology; viii) orthopaedics & traumatology; ix) surgery; x) ear, nose & throat and xi) paediatrics medicine.

This study was undertaken in April, 2012, with the approval of the Institutional Review Board of the University of Hong Kong/Hospital Authority Hong Kong West Cluster (IRB UW-12-161).

#### Data analysis

Data collected from the student survey, including the students’ demographic information, attitude score and awareness, were entered into Microsoft Excel 2007 and analysed with IBM SPSS 22.0 (SPSS Inc., Chicago, USA). The students’ attitude scores were calculated from their answers. Each “Yes” and “No” answer was given 1 and 0 mark respectively for question 7 to 14. This would add up to a sum which ranged from 0 to 8 marks in total. The students’ attitude scores were categorized into three groups which were poor (scored 0–2), average (scored 3–5) and good (scored 6–8). Descriptive analysis and analytical statistics were employed in the data handling. The independent variables were curriculum, year of study, age, gender, regular family doctor, and regular dentist. Analysis of variance (ANOVA) was used to explore the influence of the six independent variables on the students’ attitude scores. Logistic regression was performed to study the correlation of the independent variables with student awareness. The statistical significance level for all tests was set at 5%.

## Results

This survey targeted all 1,857 medical students and dental students from the two universities. A total of 809 valid questionnaires were collected from 577 medical students and 232 dental students. The response rate was 44%. Those who returned a blank questionnaire were not considered as participating.

Table 1 shows the participating students’ profiles and concurrence of items relating to their attitude to collaboration between medical and dental practice. Most students (97%) agreed that oral health is an integral part of general health, but many did not agree that medical students should have a rotation in dentistry (42%) or vice versa (36%). The majority of the students (n = 556, 75%) had good attitude (score 6–8) toward to collaboration between medical and dental practice and only a few (n = 8, 1%) had poor attitude (score 0–2). The mean attitude score (SD) towards the collaboration derived from these 8 items was 6.37 (1.44). There is a significant difference in the mean attitude score between respondents who did (n = 426) or did not agree (n = 311) that medical students should have a rotation in dentistry (7.13 ± 1.03 vs. 5.31 ± 1.26; p < 0.001).

**Table 1:** **Participants’ profile and concurrence of items related to attitude towards collaboration between medical and dental practice**

**Item (No. of respondents)**	**Group**	**No. (%)**
1. Curriculum (n = 809)	Medicine	577 (71%)
	Dentistry	232 (29%)
2. Year of Study (n = 809)	Year 1–3	561 (69%)
	Year 4–5	248 (31%)
3. Age (n = 796)	20 or below	345 (44%)
	21 or above	451 (56%)
4. Gender (n = 796)	Male	419 (53%)
	Female	377 (47%)
5. Regular family doctor (n = 797)	Yes	323 (40%)
	No	474 (60%)
6. Last dental check-up (n = 797)	Within 1 year	408 (51%)
	Over 1 year	489 (49%)
7. Dentist is a profession similar to medical practitioners (n = 799)	Yes	735 (92%)
	No	64 (8%)
8. Oral health is an integral part of general health (n = 799)	Yes	774 (97%)
	No	25 (3%)
9. Dentists should be included in electronic health record systems (n = 796)	Yes	671 (84%)
	No	125 (16%)
10. Medical-dental collaboration enhances quality of patient care (n = 787)	Yes	629 (80%)
	No	158 (20%)
11. Dentist is responsible to advise patient on systemic health (n = 775)	Yes	575 (74%)
	No	200 (26%)
12. Physician is responsible to advise patient on oral health (n = 794)	Yes	688 (87%)
	No	106 (13%)
13. Dental students should have a rotation in medicine (n = 801)	Yes	509 (64%)
	No	292 (36%)
14. Medical students should have a rotation in dentistry (n = 801)	Yes	462 (58%)
	No	339 (42%)
15. Aware of any collaboration between dentistry and medicine (n = 801)	Yes	616 (77%)
	No	185 (23%)

Table 2 shows students’ attitudes towards collaboration between medical and dental practice and the six variables. Curriculum, regular dentist, and gender were significant factors that affected student attitude (Table 3). The males had a more positive attitude about the collaboration than female students (β = 0.235, p = 0.027). Students studying dentistry were more positive than those studying medicine (β = 0.651, p < 0.001) and regular dental attenders were more so than irregular dental attenders (β = 0.263, p = 0.014).

**Table 2:** **Students’ attitudes towards collaboration between medical and dental practice and variables**

**Independent variables**	**Group (n)**	**Mean (SD)**	**p value**
Curriculum	Medicine (577)	6.18 (1.50)	<0.001*
	Dentistry (232)	6.82 (1.19)	
Last dental checkup	Within 1 year (408)	6.50 (1.42)	0.005*
	Over 1 year (489)	6.02 (1.46)	
Gender	Male (419)	6.46 (1.46)	0.077
	Female (377)	6.27 (1.14)	
Regular family doctor	Yes (323)	6.30 (1.46)	0.319
	No (474)	6.41 (1.44)	
Year of study	Year 1–3 (561)	6.39 (1.45)	0.361
	Year 4–5 (248)	6.29 (1.43)	
Age	20 or below (324)	6.40 (1.45)	0.619
	21 or above (411)	6.35 (1.43)	

*Significant < 0.05.

**Table 3:** **Final model (ANOVA) of the students’ attitudes towards collaboration between medical and dental practice and variables**

**Variable**	**Parameter estimate (B)**	**S.E.**	**p value**
Curriculum			
Dentistry	0.651	0.116	<0.001
Medicine^a^			
Last dental check-up within 1 year			
No	−0.263	0.107	0.014
Yes^a^			
Gender			
Female	−0.235	0.106	0.027
Male^a^			
(Intercept)	6.812	0.376	<0.001

^a^Reference category; Adjusted R^2^ = 0.055.

**Figure 1:**
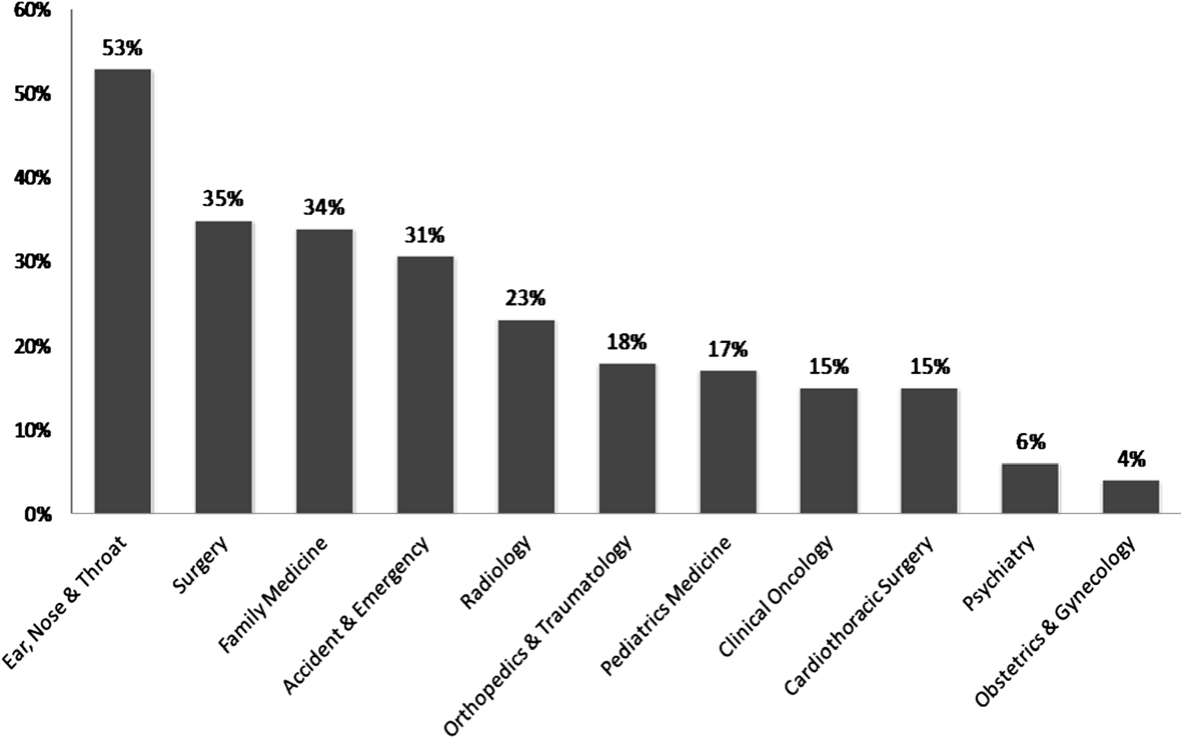
Students’ perception (% respondents) on the medical disciplines that relate to dentistry.

Most students (n = 616, 77%) were aware of collaboration between medical and dental practice in Hong Kong (Table 1). Table 4 shows students’ awareness towards collaboration between medical and dental practice and the 6 variables. The results of the logistic regression (Table 5) show that dental students were more aware of the collaboration between medical and dental practice than medical students (OR: 4.38, 95% C.I.: 2.65–7.25; p < 0.001). Seniors (year 4 or 5) were more aware of the collaboration than juniors (OR: 1.76, 95% C.I.: 1.18–2.62; p = 0.005). Many students recognized that dentistry has a close link to ear, nose & throat but few recognized its link to obstetrics & gynaecology (Figure 1).

**Table 4:** **Students’ awareness towards collaboration between medical and dental practice and significant variables**

**Independent variable**	**Group (n, %)**	**p value**
Curriculum	Medicine (407, 71%)	<0.001*
	Dentistry (212, 91%)	
Year of study	Year 1–3 (411, 73%)	0.001*
	Year 4–5 (208, 84%)	
Age	20 or below (253, 73%)	0.078
	21 or above (355, 79%)	
Gender	Male (325, 78%)	0.452
	Female (283, 75%)	
Regular family doctor	Yes (254, 79%)	0.440
	No (361, 76%)	
Last dental check-up	Within 1 year (316, 78%)	1.000
	Over 1 year (301, 77%)	

*Significant < 0.05.

**Table 5:** **Final model (logistic regression) on the students’ awareness towards collaboration between medical and dental practice and significant variables**

**Variable**	**Odds ratio**	**95% CI**	**p value**
Curriculum:			
Dentistry	4.380	2.646–7.246	<0.001
Medicine^a^			
Year of Study:			
Year 4–5	1.763	1.84–2.624	0.005
Year 1–3^a^			
Constant	3.644		0.001

^a^Reference category.

## Discussion

This study is a questionnaire survey to evaluate the attitude and awareness of medical and dental students toward collaboration between medical and dental practice in Hong Kong. The questions used in this study were adapted from the questions used by Hendricson and Cohen [11] and Migliorati and Madrid [12]. The questions of the 2 studies were wide-ranging in medical-dental collaboration and education. We selected specific questions that related to the collaboration between medical and dental practice. A one page closed-ended questionnaire was employed and would be quick for the students to answer [13]. The answers of different respondents would also be easy to compare and analyse statistically. However, the questions can force respondents to give simplistic responses to ‘complex’ questions and, therefore, interpretation of the results must be taken with caution.

Inviting the students to join this study by email would have been a low-cost and convenient method, but was not used because it generally leads to a low response rate [14]. Instead, the questionnaires were distributed directly after the lesson in the lecture theatre and collected immediately after completion. A study found this method is efficient and effective for collecting responses from students [15]. However, 56% of the students still did not participate in this study. One reason was that the fourth and final year medical students were on their clinical rotation period in small groups. Not all could be contacted in their residential halls and, therefore, could not respond to the questionnaire. This response rate may undermine the power of the study. The outcome of this study cannot, therefore, be taken to be conclusive. Without the characterization of the non-respondents, the study might suffer from response bias as participants may simply be those who have a positive disposition to the study objective. Therefore, results should be interpreted with caution.

In this study, the medical and dental students generally demonstrated good attitude to collaboration between medical and dental practice. Most students agreed that oral health is an integral part of general health and that dentist is a profession similar to medical practitioners. However, more than a third of the respondents did not agreed with the medical-dental rotation, which Hendricson and Cohen consider not only be beneficial but essential [11]. Gender, regular dental check-up, and curriculum were associated with students’ attitudes. The results agreed with a previous study that reported that gender could affect a student’s attitude towards medical-dental collaboration [16]. Studies found preventive health behaviours such as attending regular dental check-ups correlated with good attitudes towards learning [17,18]. This good attitude towards learning could be associated with a good attitude to collaboration between medical and dental practice. Curriculum was also found to be associated with students’ attitudes towards and awareness of collaboration between medical and dental practice. In Hong Kong, the dental curriculum emphasises problem-based learning, whereas the medical curriculum is a hybrid between problem-based and discipline-based learning. Dental students may have more opportunities to develop communication skills that encourage collaboration. This difference in curriculum may have affected the measured attitudes and awareness, since studies have shown that case-based learning can enhance the effectiveness of inter-professional collaboration [19,20].

Greater awareness of collaboration between medical and dental practice was found among senior students (year 4 and 5) than junior students (years 1–3). This may be explained by the level of education and age, which have a positive correlation with maturity [21]. Furthermore, senior students have more exposure to clinical care and teamwork, which may have led to better awareness to collaboration between medical and dental practice. This study found students were most aware of the medical disciplines of ENT and general surgery in the collaboration between medical and dental practice. This might be due to the proximity of the oral cavity to the organs and the nature of the medical discipline to dental surgery. This finding implied the students did not have a complete understanding of the depth of the collaboration between medical and dental practice. Once the depth of that link is understood, the need for collaboration between medical and dental practice by both professionals would be clearer. Students should realize collaboration between medical and dental practice can involve any discipline not just pertaining to those related to the oral cavity. Dentists should identify their patient’s need of primary prevention strategies for cardiovascular disease [22]. Patients with common systemic conditions, such as diabetes mellitus and pregnancy, often experience dental complications.

Interprofessional continuing education is a useful means of breaking down stereotypes about another professional’s identity, improving teamwork in clinical practice [23]. The president of the Hong Kong College of Dental Surgeons has stated that physicians and dentists should have access to updated knowledge about the collaboration between medical and dental practice and that there should be guidelines and methods to increase confidence in providers’ ability to identify and appropriately refer patients who have diseases [8]. It is important to enhance health care services through close collaboration between medical and dental professionals. Continuing education courses should emphasize the importance of interprofessional collaboration as and include methods to increase confidence in providers’ ability to identify and appropriately refer patients with disease [24]. Medical-dental collaboration can be facilitated by medical and dental professional bodies, which should develop guidelines for the indications, timing, protocols, and responsibilities of referral and consultation among physicians and dentists. Public awareness should also be aroused so that patients and the community alike understand the relationship between oral and systemic health. In doing so, a better understanding of collaboration in the community may benefit all parties with improved professional cooperation and a better education of the public.

## Conclusion

In this study, medical and dental students demonstrated a good attitude towards and awareness of collaboration between medical and dental practice in Hong Kong. This establishes an essential foundation for continually fostering collaboration, which is vital to improving resource efficiency and the standard of care.

## Additional file

Additional file 1:
**Questionnaire to the medical and dental students.**
