# Supplementary material for: Attitude and awareness of medical and dental students towards collaboration between medical and dental practice in Hong Kong
Source: BMC Oral Health. 2015 May 2;15:53. doi: 10.1186/s12903-015-0038-2 (PMC4426176; doi:10.1186/s12903-015-0038-2)
Supplement: Additional file 1: — Questionnaire to the medical and dental students. [file 12903_2015_38_MOESM1_ESM.docx]

**Additional file 1 Questionnaire to the medical and dental students**

1. Program: Medicine Dentistry

2. Year of study: Year 1 to 3 Year 4 or 5

3. Age: Below 21 21 and above

4. Gender: F M

5. Do you have a family doctor (your personal physician)?

Yes No

6. Was your last *regular* dental check-up within 1 year?

Yes No

7. Do you perceive dentist as a professional similar to medical practitioner?

Yes No

8. Do you view oral health as an integral part of general health?

Yes No

9. An electronic health record system may develop to allow the private and public medical sectors to share patient information. Do you think it is necessary to include dentists into this system?

Yes No

10. Do you think it is essential to develop collaboration between medical and dental practice for patient care?

Yes No

11. If you were a dentist, do you feel responsible to advise your patient about general health issues?

Yes No

12. If you were a physician, do you feel responsible to advise your patient about oral health issues?

Yes No

13. Do you think dental students should have a rotation in family medicine at the hospital where they can work on patients in conjunction with medical students?

Yes No

14. Do you think medical students should have a rotation in oral diagnosis screening clinic to work on patients in conjunction with dental students?

Yes No

1. 15. Are you aware of any collaboration between dentistry and medicine?
2. Yes No

16. If yes, which discipline(s) have you seen collaboration between medical and dental practice?

(You may indicate more than one discipline)

Accident & Emergency Services Family Medicine Psychiatry

Cardiothoracic Surgery Obstetrics &Gynecology Radiology

Clinical Oncology Orthopedics & Traumatology Surgery

Ear, Nose & Throat Paediatrics Medicine
